# Artificial Intelligence for developing a personalised functional profile and supporting upper-limb rehabilitation decision-making in children with unilateral Cerebral Palsy (AInCP): protocol for an observational study

**DOI:** 10.1136/bmjopen-2026-117833

**Published:** 2026-11-02

**Authors:** Giuseppina Sgandurra, Alexandra Kalkantzi, Elena Beani, Giovanni Cioni, Rocio Palomo Carrión, Hilde Feys, Nana Nino Tatishvili, Matteo Cianchetti, Fiorella Battaglia, Giuseppe Turchetti, Wil Van Dommelen, Marco Pirini, Francesca Fedeli, Clara Bombonato, Maria Chiara Di Lieto, Benedetta Del Lucchese, Veronica Barzacchi, Valentina Menici, Annalisa Cotardo, Marta Rizzo, Eleonora Bieber, Pietro Ilio Gatti, Federica Camuncoli, Michela Tosetti, Paolo Bosco, Laura Biagi, Luca Peretti, Francy Cruz-Sanabria, Cristina Lirio-Romero, María Coello-Villalón, Purificación López-Muñoz, Julián Ángel Basco-López, Helena Romay Barrero, Lisa Mailleux, Sofia Tatishvili, Anna Bedoshvili, Nino Valishvili, Martina Maselli, Giovanna De Luca, Corrado Claverini, Leopoldo Trieste, Giorgio Manfroni, Harold Bult, David Berghold, Iris Jakob, Alex Michael Pagnozzi, Jurgen Fripp, Kerstin Pannek, Els Ortibus, Antea Scrocco, Gemma D’alessandro, Giovanni Arras, Tommaso Turchi, Paolo Ferragina, Roslyn N Boyd, Silvia Filogna, Giuseppe Prencipe

**Affiliations:** 1 Department of Clinical and Experimental Medicine, University of Pisa, Pisa, Italy; 2 Department of Neurodevelopmental Neuroscience, IRCCS Fondazione Stella Maris, Calambrone, Italy; 3 Department of Rehabilitation Sciences, KU Leuven, Leuven, Belgium; 4 Child and Youth Institute, KU Leuven, Leuven, Belgium; 5 Nursing, Physiotherapy and Occupational Therapy Department, Universidad de Castilla-La Mancha, Toledo, Spain; 6 David Tvildiani Medical University, Tbilisi, Georgia; 7 The BioRobotics Institute, Scuola Superiore Sant’Anna, Pisa, Italy; 8 Department of Humanities, University of Salento, Lecce, Italy; 9 Institute of Management, Scuola Superiore Sant’Anna, Pisa, Toscana, Italy; 10 Noldus group, Wageningen, The Netherlands; 11 Khymeia Group, Padova, Italy; 12 FightTheStroke Foundation, Milano, Italy; 13 Laboratory of Medical Physics and Magnetic Resonance, IRCCS Fondazione Stella Maris, Calambrone, Italy; 14 Tyromotion GmbH, Graz, Austria; 15 Queensland Cerebral Palsy and Rehabilitation Research Centre, Child Health Research Centre, The University of Queensland, Brisbane, Queensland, Australia; 16 The Australian e-Health Research Centre, Commonwealth Scientific and Industrial Research Organisation (CSIRO), Brisbane, Queensland, Australia; 17 Department of Development and Regeneration, KU Leuven, Leuven, Flanders, Belgium; 18 Cerebral Palsy Reference Centre, University Hospitals Leuven, Leuven, Flanders, Belgium; 19 Department of Neuroscience, Psychology, Drug Area and Child Health (NEUROFARBA), University of Florence, Florence, Italy; 20 Department of Computer Science, University of Pisa, Pisa, Italy

**Keywords:** Artificial Intelligence, Family, Developmental neurology & neurodisability

## Abstract

**Introduction:**

Cerebral palsy (CP) is a lifelong non-progressive disorder, resulting from disturbances in the developing or infant brain, with approximately 17 million people worldwide being diagnosed. More than one-third of the population with CP has unilateral CP (UCP), whereby the primary sensorimotor impairments affect predominantly one side of the body, mainly the upper limb (UpL), compromising independence and quality of life. Associated comorbidities (eg, cognitive, visual, behavioural impairments and epilepsy) can impede the establishment of a clear functional diagnosis, consequently compromising the formation of effective intervention strategies. Driven by the clinical presentation complexity of UCP, the main objective of the Artificial Intelligence in Cerebral Palsy (AInCP) study is to establish a valid functional diagnosis supporting accurate rehabilitative priorities, identifying multidimensional functional profiles that may inform personalised rehabilitation planning, utilising AI. Through the integration of multimodal data, the ultimate goal is to develop and test evidence-based clinical decision support tools for the personalised functional diagnosis and UpL assessment in children with UCP by developing, testing and validating AI-cost-effective strategies. This will enable more accurate, personalised functional diagnoses and treatment planning, enhancing quality of life and efficiency of care for children with UCP.

**Methods and analysis:**

This prospective, multicentre, observational clinical study will include 250 children with UCP and 250 typically developing children (TDC). Participants will be enrolled and assessed within four clinical centres distributed across Europe. Data from children with UCP will be collected by three broad domains: clinical assessments and questionnaires, neuroimaging data and data from UpL daily-life monitoring using wearable sensors. TDC will be evaluated using clinical tests and wearable sensors. All the items identified and the whole methodology have been co-created and co-designed among a multidisciplinary team, including caregivers and persons with lived experience. Data will be analysed with AI and machine-learning algorithms to understand the interdependencies across functional domains and create functional profiles that can guide personalised treatment planning. Non-clinical data such as sleep quality, technology familiarity and technology acceptance will also be collected to provide an empirical foundation for conducting an early health technology assessment of the solutions developed within the AInCP project.

**Ethics and dissemination:**

Ethical approval has been obtained by all involved clinical sites. Written informed consent will be obtained from the legal representatives of all participating children, and age-appropriate assent will be obtained from children according to local ethical requirements. All procedures will be closely monitored by ethicists during the implementation of AI to ensure patient privacy, prevent bias and avoid unintended harm while developing robust, fair and trustworthy AI solutions. The results of this study will be disseminated through open-access peer-reviewed journals relevant for paediatrics to ensure wide accessibility. Results will be communicated by the professionals directly involved in the clinical study through national and international academic conferences, relevant associations and organisations, including policy makers.

**Trial registration number:**

NCT06073522.

**STRENGTHS AND LIMITATIONS OF THIS STUDY**

- The study involves implementation of a wide range of clinical, neuroimaging and technological data to provide a detailed functional diagnosis (functional profiling).
- Involvement of four clinical centres across Europe (including both high-income and low–middle-income countries) will enhance the generalisability of the findings.
- The study involves a multi-disciplinary group including clinicians, engineers, ethicists, economists and families/persons with lived experience to achieve a holistic approach.
- Utilisation of advanced artificial intelligence techniques for data analysis aims to improve the accuracy and personalisation of the functional diagnosis.
- Limitations include potential bias due to MRI acquisition from different scanners and technical challenges with equipment (ie, wearable sensors) that could affect data collection.

## Introduction

 Cerebral palsy (CP) is the most common cause of childhood chronic physical disability,^1^ with approximately 17 million individuals being diagnosed worldwide,^2^ although some broader or more recent reports round the figure upward slightly (eg, to 18 million) to account for underreported cases, especially in low-income and middle-income countries. One of the most frequent CP subtypes, accounting for more than one-third of the total CP population, is unilateral CP (UCP), whereby the sensorimotor impairments affect predominantly one side of the body, with the upper limb (UpL) being generally more affected than the lower limb. From a functional perspective, children with UCP typically fall within levels I–III of the Manual Ability Classification System (MACS), as the presence of a fully functional, non-involved hand generally prevents the need for the continuous and extensive assistance characteristic of MACS levels IV and V. A large heterogeneity in UpL deficits has been reported among children with UCP, with common manifestations including difficulties in performing daily UpL activities such as reaching, grasping, releasing and manipulating objects. Along with the motor impairments such as reduced muscle length, increased muscle tone and weakness,^3^ associated comorbidities such as somatosensory and visual impairments, decreased cognitive ability or behavioural challenges may be present.^4–6^ Contextual factors, including the child’s personal characteristics (eg, personality, motivation and interest in the activity) as well as environmental factors (eg, parental support, daily routine and social environment), may further affect the level of functionality.^7 8^ These limitations in overall functioning might restrict not only independence and daily function but also reduce their participation in natural contexts (ie, home, school and broader community life).

Stating this complexity, the assessment of various domains of the child’s functioning has a key role in defining the clinical picture and create tailored interventions, maximising functional possibilities on a personal and community level.^9^ The clinical assessment of children with UCP is traditionally provided by a set of different functional classification systems, clinical tests and questionnaires joined with brain MRI to evaluate the complex aspects of UpL function, brain structure, lesion severity and functional relationships. Currently, functional diagnosis and the consequent rehabilitative approach in children with UCP are based on the aforementioned combination, with the absence of available tools to identify and classify the multiphenotypic aspects of UCP. The spontaneous use of the UpL is therefore crucial to acquire a precise functioning profile in daily-life within a natural setting, with the only tools currently available being self-reported questionnaires, while wearable trackers have been proposed to monitor the spontaneous activities of the UpL in a natural environment.^10 11^ Although clinical assessment, neuroimaging and wearable trackers are important for the clinical phenotyping of UpL functioning of children with UCP in daily life, they have not been previously analysed together. Having the possibility to combine and match the results coming from different areas could improve the diagnosis of children with UCP and build a patient-specific profile.^12^ In this respect, the application of artificial intelligence (AI) techniques is enabling new frontiers in the prediction of the motor outcome of UCP^13^ by allowing data integration ranging from traditional clinical assessments to wearable devices. AI allows examination of large and complex datasets, defined as ‘Big Data’ to uncover hidden patterns, unknown correlations and other useful information that can be used to make prevision or better decisions.^12 13^ In particular, AI-based techniques empower the analyses of Big Data or digital biomarkers taking into account what can be learnt from the data itself. Various tools and technologies are used, including data mining, machine-learning^14^ and deep-learning algorithms to automate the process of extracting insights from large datasets and identify patterns that may be missed by traditional statistical analyses. In the clinical scenario, AI methods can combine smart tools and algorithms to provide more accurate diagnosis, guide rehabilitation strategies and consequently improve patient outcomes by detecting changes in health conditions, suggesting and giving access to a new way for assisting clinicians in the decision-making process.^15^

The overall rationale for this prospective, observational, clinical study is to identify, collect and combine through AI and Big Data analysis multidimensional biomarkers to provide an accurate tool for functional diagnosis in children with UCP. This will be pursued by collecting a broad array of data, including clinical assessments, brain neuroimaging and daily-life UpL monitoring. By analysing data from multiple domains and accounting for their interdependencies, functional diagnosis can become more individualised, fostering the formation for tailored treatment strategies. Such a multidimensional approach has not only clinical but also scientific and economic implications.

## Methods and analysis

### Aims and hypotheses

The overall aim of this study is to develop a diagnostic decision support tool (dDST) to provide a valid and reliable functional diagnosis for the UpL of children with UCP, enabling accurate stratification based on individual characteristics. The main objective is to identify, collect and combine relevant biomarkers (ie, clinical multiaxial assessments, neuroimaging and UpL daily movement analysis) within a comprehensive assessment protocol. This can be achieved by understanding the relationship between the data and the distinct clinical features that differentiate each participant from another.

The analysis will point towards the most important biomarkers that can create personalised profiles that delineate the clinical and functional picture of each child with UCP, informing clinicians about each individual child’s functional abilities and identifying current rehabilitation needs that could be considered for a personalised treatment plan.

A further aim will be to evaluate, by means of questionnaires, the different stakeholders’ point of view, such as early acceptability, usability, quality of life and simulating the related potential sustainability, that is, cost-effectiveness and cost-utility of the AInCP approach, if compared with the standard of care. This is part of an early multistakeholder, multidimensional assessment of the potential value of AInCP solutions within an early HTA (eHTA) approach.

### Study design and setting

This prospective, multi-centre observational clinical study will take place at four clinical centres in Italy (IRCCS Fondazione Stella Maris), Spain (University of Castilla-La Mancha (UCLM)), Belgium (KU Leuven) and Georgia (David Tvildiani Medical University (DTMU)). The enrolled participants will be evaluated only at one time-point in accordance with a standardised assessment protocol across all four clinical centres. In addition, a part of neuroimaging data will be analysed by the Laboratory of Medical Physics and Magnetic Resonance of IRCCS Fondazione Stella Maris and the Australian e-Health Research Centre and the Commonwealth Scientific and Industrial Research Organisation (CSIRO) (with a specific ethics approval by CSIRO Health and Medical Human Research Ethics Committee (2022_047_LR).

### Participants

Children with UCP and typically developing children (TDC) aged 5–15 years will be included in this study. Specific criteria are established for the groups involved.

For the UCP group, the inclusion criteria comprise the following: (1) confirmed diagnosis of UCP, predominantly spastic type of motor disorder and (2) manual ability levels from I to III based on the MACS.^16^ The exclusion criteria are as follows: (1) severe UpL impairment (MACS ≥level IV, inability to grasp), (2) botulinum toxin-A injections in UpL within 6 months prior to study entry, (3) UpL surgery within 12 months prior to study entry and (4) severe comorbidities and/or severe cognitive disability prohibiting sufficient cooperation (ie, unable to undertake the assessment protocol). For the TDC group, the inclusion criterion will be the absence of documented clinically relevant disorders.

Before the assessment of the enrolled participants with UCP, a screening of all clinical measurements included in the AInCP clinical protocol, which are traditionally carried out for routinely clinical purposes, will be undertaken to establish the best time window for the assessment. If certain cognitive tests have been administered within the last 6–12 months prior to the established timepoint assessment, the results and all the detailed scores will be requested. If this is not possible, the assessment will be rescheduled to avoid two close executions of the same tests or potential learning effects because of test-retest.

The selection of participants and evaluation process of all participants will be carried out by the research/clinical staff of the four clinical centres: the primary evaluation settings consist of the relative institutions (ie, universities and clinical research hospitals) and, if necessary, other contexts such as community centres, schools or home.

### Informed consent for study participation

Informed consent forms are developed to ensure that participants are fully aware of the nature, purpose and potential risks and benefits of the study before agreeing to participate. For this project, the informed consent form is officially signed by the legal caregiver, as the participants are minors.

Informed consent forms for the parents of both participants with UCP and TDC have been developed. The documents include the rationale and overall aim of the project, assessment protocol described in detail, legal aspects, insurance policy, data protection and retention policy and contact information of the researchers. Further, two versions of age-appropriate documents were created: an information sheet for children aged 5–12 years and an informed assent for children aged >12 years in accordance with the Declaration of Helsinki. Lastly, a third version of informed consent form was developed for the clinicians following up the participants with UCP. Based on the AInCP protocol, these healthcare providers will fill in part of the early eHTA questionnaire to assist in understanding the potential benefit of technologies in the rehabilitation regimen and potential modifications that are required.

### Personalised functional profile

The AInCP protocol envisages a thorough evaluation of children with UCP, which consists of a diverse array of assessments (figure 1).

**Figure 1:**
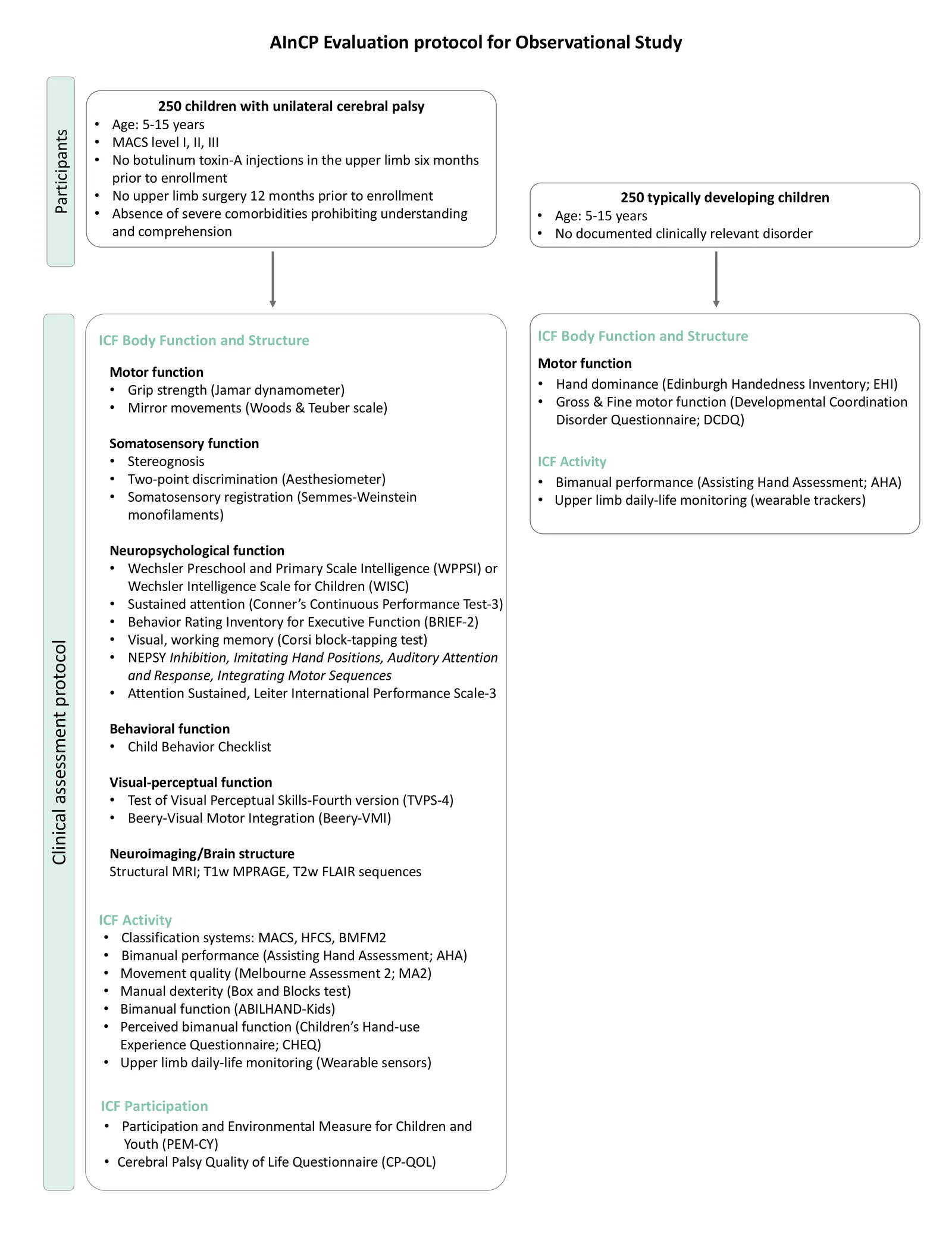
Flowchart documenting the clinical evaluation protocol of the AInCP study. AInCP, Artificial Intelligence in Cerebral Palsy; BMFM2, Bimanual Fine Motor Function, Version 2; BRIEF-2, Behavior Rating Inventory of Executive Function, Second Edition; FLAIR, fluid-attenuated inversion recovery; HFCS, House Functional Classification System; ICF, International Classification of Functioning, Disability and Health; MACS, Manual Ability Classification System; MPRAGE, magnetisation-prepared rapid acquisition with gradient echo; NEPSY, A Developmental Neuropsychological Assessment.

Classification systems will be used to describe the overall functioning of UpL in children with UCP. The definition of the classification level will be agreed among the clinicians and the child’s caregivers or legal guardians. First, *MACS *will be used to describe the global ability to handle objects in daily activities on one of five levels.^16^ In line with the data reported in literature and in accordance with the inclusion and exclusion criteria, all enrolled participants will be classified in MACS levels: I (ie, able to handle objects easily and successfully), II (ie, able to handle most objects but with somewhat reduced quality and/or speed of achievement so that alternate ways of performance might be used) or III (ie, handles objects with difficulty; needs help to prepare and/or modify activities). Second, the *House Functional Classification System (HFCS*) will be used to define the UpL function of the affected hand on the basis of hand use and grasp ability, ranging from ‘does not use’ (level 0) to ‘spontaneous use, complete’ (level 8).^17^ Lastly, the *Bimanual Fine Motor Function-version 2 (BFMF 2*) will classify the functioning of the two hands on a five-level scale, whereby level I describes the best and level V the most limited function.^18^

Then, the three broad domains in which the assessments can be grouped are as follows:

- Clinical assessment tools and questionnaires
- Brain neuroimaging
- UpL daily-life monitoring

The selected assessments cover the target age range (ie, 5–15 years) and have been developed either specifically for children with CP or are being routinely used in this population. In addition, psychometric properties were considered for the development of the assessment protocol.

#### Clinical multiaxial assessments and questionnaires

##### Upper limb neuromotor factors assessments

Neuromotor factors, as a direct consequence of the brain lesion, will be evaluated.^19^ First, UpL muscle tone will be evaluated using the *Modified Ashworth Scale (MAS*).^20^ Resistance in passive movement is graded on a scale from 0 to 4, with an additional degree of +1. Grip strength will be assessed with the *Jamar hydraulic dynamometer* based on the review of Dekkers *et al.*^21^ Three trials will be performed for each hand: first the non-dominant hand will be assessed, and the mean of trials per hand will be recorded. Lastly, mirror movements will be evaluated using the *Woods and Teuber scale*,^22^ through the performance of three hand tasks: (1) fist opening and clenching, (2) finger opposition and (3) finger tapping.^23^

##### Upper limb somatosensory assessments

Somatosensory function will be assessed on the basis of two modalities: tactile registration and tactile perception. Tactile registration will be evaluated using the *Semmes–Weinstein monofilaments*. The evaluation starts with the 0.07-gram monofilament reflecting normal tactile registration. Based on the participant’s performance, the use of a lighter (more difficult) or heavier (easier) monofilament will follow.^24^ Then, tactile perception will be evaluated via the assessment of stereognosis and two-point discrimination. For stereognosis, *12 daily-life objects* (six paired and six unpaired) will need to be identified by the participant with the vision occluded. For two-point discrimination (ie, the ability to discern that two nearby objects touching the skin are truly two distinct points, not one), an *aesthesiometre* will be used, which can determine the minimum distance that the two impressions can be distinguished by the child.^25^ The aforementioned assessments will be administered in accordance with the protocol of Klingels *et al.* ^25^

##### Upper limb movement skills assessment

A series of assessments will be used to evaluate UpL activity. First, the *Assisting Hand Assessment (AHA*) will evaluate bimanual performance, defined as the spontaneous use of the affected hand in bimanual tasks. The AHA is the gold standard for assessing bimanual performance and represents the primary outcome measure in the AInCP protocol. Established psychometric properties involve excellent internal scale validity (intraclass correlation coefficient (ICC), 0.98),^26^ interrater (ICC, 0.97) and test–retest reliability (ICC, 0.99).^27^ The AHA assessment session comprises a playful session involving semi-structured activities calibrated on the different ages.^28^ The assessment is videotaped, and the scoring is based on the video. In this protocol, the AHA will be administered with the addition of two wearable sensors on the two wrists of the child, as described below. This addition to the test will assist in interpreting the movement patterns/trajectories as recorded by the wrist sensors. Thereafter, UpL movement quality will be assessed using the *Melbourne Assessment –* 2, a valid and reliable criterion-referenced test, consisting of 14 unimanually-tested items including reaching, pointing, manipulation, grasp, release and pronation/supination.^29^ Lastly, manual dexterity will be evaluated using the *Box and Blocks Test*. Participants will be requested to transfer 150 blocks from one side of a rectangular box to the other, as fast as possible within 60 s. Each hand will be evaluated separately, starting from the less-affected side.^30^

##### Cognitive and neuropsychological assessments

Cognitive profile will be evaluated using either the *Wechsler Preschool and Primary Scale of Intelligence – fourth edition (WPPSI-IV*) or the *Wechsler Intelligence Scale for Children – fourth or fifth edition (WISC-IV or WISC-V*),^31 32^ depending on the age of the children (pre-school or school ages, respectively). The latest version available in each clinical partner’s country will be used.

In addition, a battery of neuropsychological tests has been planned to assess attention, executive functions and visuo-motor planning and integration. Concerning attention processes, the *Conner’s Continuous Performance Test (CPT-3*) will be administered to evaluate four dimensions of attention: inattentiveness, impulsivity, sustained attention and vigilance.^33^ This computerised test, based on a go–nogo task, requires participants to respond to a series of visual stimuli, responding when they see a target stimulus and inhibiting their response when they see a non-target stimulus. Across the different dimensions which it assesses, the CPT-3 has demonstrated test–retest reliability reaching up to 0.84 of ICC.^34^ Additionally, the *Sustained Attention subtest of the Leiter International Performance Scale* will be used to assess visual attention through repetitive barrage tasks performed within a predefined time.^35^ The *Auditory Attention subtest of the NEuroPSYchological assessment (NEPSY-II*) will also be used, requiring the children to touch specific-coloured shapes when they hear the corresponding names and to remain still when they hear other words, including distractors represented by other colours.^36^

Regarding executive functions, the *inhibition* subtest of NEPSY-II will evaluate the ability to inhibit automatic responses in favour of novel responses and to switch between response types. It is divided into three conditions: naming, inhibition and switching, with both accuracy and speed recorded for each condition. The *response set subtest* of NEPSY-II will assess the ability to shift and update new and complex sets of rules, involving the inhibition of previously learnt responses. The v*erbal fluency subtes*t of NEPSY-II evaluates cognitive flexibility in a word production task within a specific semantic or phonemic category.^36^ The *Corsi block-tapping test* will evaluate visuospatial short-term and working memory.^37^ The *Behaviour Rating Inventory of Executive Function*, a parent-reported questionnaire, examines everyday behaviour associated with executive functions, including planning, focusing attention, remembering instructions, inhibition, flexibility and working memory.^38^

The *Beery Test of Visual-Motor Integration (Beery-VMI*), a paper-pencil test, will be used to determine visuomotor integration abilities.^39 40^ The *imitating hand positions and manual motor sequences subtests* of NEPSY-II will assess motor planning and coordination abilities, requiring the child to imitate various hand/finger positions performed by the examiner with either the dominant or non-dominant hand, and a series of rhythmic unimanual or bimanual movements shown by the examiner.^36^ Lastly, visual-perceptual function will be evaluated using the *Test of Visual Perceptual Skills (TVPS-4*), a motor-free and individually administered test with the following subscales: visual discrimination, visual memory, spatial relationships, sequential memory, figure-ground, visual closure and form constancy.^41 42^

##### Clinical questionnaires

Clinical questionnaires will be administered to complete the assessment of overall functioning. Based on the domain of interest, the following questionnaires have been selected: the *ABILHAND-Kids questionnaire* (reliability R, 0.94; reproducibility R, 0.91), evaluating manual abilities of children with CP, with a particular reference to the independence in achieving daily-life tasks (ie, open a jar with jam and buttoning a shirt), by exploring if some activities are easily performed, if they present some difficulties or if they are impossible.^43^ Further, the 27-item *Children’s Hand-use Experience Questionnaire (CHEQ*), freely available via the website (www.cheq.se), is designed to measure children’s experiences when using the affected hand in common daily-life activities requiring use of both hands. For CHEQ, three scales are used to measure grasp efficacy when both hands are involved, time spent when performing the activity compared with peers and experience of feeling bothered while performing the activities independently.^44^

The *Participation and Environmental Measure for Children and Youth*, a parent-report measure, evaluates the participation and environment of the child across three settings: home, school and community.^45 46^ The specific *Cerebral Palsy Quality of Life Questionnaire (CP-QOL*) assesses all domains of life quality, including social well-being and acceptance, functioning, participation, physical health, emotional well-being, access to services, pain, impact of disability and family health. Specific different forms will be adopted for children (9–12 years), teenagers (13–18 years) and parents (4–18, with the two versions for parents of children aged 4–12 years or of teens 13–17 years). The answers for CP-QOL are based on a 9-point Likert scale, expressing the ‘feelings about’’ (as the degree of happiness) related to each question.^47^ Lastly, emotional and behavioural problems will also be explored through the *Child Behaviour Checklist*, completed by the parents or legal guardians.^48^

### Neuroimaging

Retrospective brain MRIs of children with UCP acquired as part of clinical care will be collected and analysed to assess the characteristics of brain lesion in children with UCP.^49^ Collected images must include a T1-weighted magnetisation-prepared rapid acquisition with gradient echo and optionally a T2-weighted fluid-attenuated inversion recovery, which are clinically available sequences. To ensure adherence to data policies and work in concert with the European General Data Protection Regulations, the images of each clinical site will be pseudonymised locally by each institution and defaced (ie, elimination of the scalp or other unique characteristics that may reveal the patient’s identity) centrally by the University of Pisa (UNIPI).

The images will be scored by expert raters using a semi-quantitative validated scale developed by Fiori *et al*.^50^ The MRI Fiori scale was specifically developed for children with CP and is a valid and reliable tool used in individuals from the age of 3 years onwards, enabling the quantification of the brain lesion severity, based on its structural appearance.^50 51^ Advanced MRI analyses will be performed on suitable data (with sufficient image quality and slice thickness <1.5 mm) using automated software that has previously been applied to a large cohort of children with UCP.^52^ This will generate quantitative measures of volume and cortical shape including cortical thickness, curvature and sulcal depth.^53 54^

### Upper limb daily-life monitoring

The assessment of UpL movements will be completed using two wearable sensors on the wrists of the children based on literature review^11^ and analysing the existing available solution. On each child’s wrist, a sensor will be worn during the AHA assessment and then for the subsequent 14 days, inside a custom-made wristband made by a soft neoprene fabric with a velcro closure. These trackers record three-dimensional UpL activity and provide the activity sum for each day. Children will be asked to wear the trackers every day for the whole day, except for night-time when the trackers shall be placed on the charger for recharging. At the end of the recording period, parents will have to place the sensors inside a box (previously received during the assessment), and they will be contacted by a member of the research staff to organise the collection of the material.

The feasibility of the use of the wearable sensors will be assessed by means of two rapid questionnaires shared with the families via the Survey Distribution Tool integrated in Research Electronic Data Capture (REDCap): the first will be the daily one, aimed to *monitor the way of use and adherence*, a fast-filling questionnaire, which will serve as a mini diary that keeps a record of special activities that the child performed or potential problems that occurred each day, during the period that the trackers are worn. The second one will be the final (ie, at the end of the 14 days of recording) and will be related to the *feasibility and satisfaction* in which families will be required to answer based on the feedback directly from the child.

### Early health technology assessment questionnaires

The set of questionnaires that will be administered during the observational study encompass a multi-dimensional multi-stakeholder approach and is dedicated to collecting information with respect to different aspects of the parents’, children’s and clinical professionals’ evaluation of the services integrated or not with the technology. Personal data and preferences such as sex, age, current occupation and other social determinants of health will be documented.

More specifically, the questionnaires include information regarding sleep quality,^55^ health-related costs, current rehabilitative service satisfaction, information and communication technologies (ICT) skills, perception of the usefulness and ease of use about general AI solutions for healthcare professionals not involved in the project (market potential). Questionnaires have been adapted for each respondent (ie, parent, child and healthcare provider) and have been translated into each respective language, following a standard translation procedure. Description of the questionnaires’ content is provided below:

The *Pittsburgh Sleep Quality Index* assesses sleep quality and disturbances over the period of the last month.^55^ It covers various aspects of sleep, including sleep duration, sleep latency, sleep efficiency, sleep disturbances, use of sleep medication and daytime dysfunction. The E*uropean Quality of Life 5 Dimensions 3 Level Version* is a standardised instrument evaluating health-related quality of life. It consists of a descriptive system that evaluates five dimensions of health (mobility, self-care, usual activities, pain/discomfort and anxiety/depression) and a visual analogue scale to rate overall health status. The Service Satisfaction Questionnaire concerns the features of current rehabilitation services that should be improved, while the scale assessing the perceived usefulness, ease of use, compatibility and usage intention of new AI-based technologies for tailored rehabilitation aims to assess potential users’ perceptions of new AI-based technologies designed for personalised rehabilitation. The ICT skills questionnaire assesses the participants’ skills and familiarity with ICT in terms of level of competence in using computers, the internet, software and other digital tools. The *health-related out-of-pocket cost issues questionnaire* focuses on the financial aspects of healthcare by exploring participants’ experiences and concerns regarding out-of-pocket expenses related to their health. Lastly, the *work-related quality of life questionnaire* evaluates life quality in relation to work and employment by assessing various aspects such as job satisfaction, work–life balance, work environment, career development and overall well-being in the workplace. Table 1 summarises the survey questionnaires and the stakeholders who will complete them.

**Table 1:** Overview of the eHTA survey questionnaires and the respective completing stakeholders

Questionnaires	Parents (UCP)	Participants (UCP)	Parents (TDC)	Participants (TDC)	Healthcare professionals	Hospital facilities
Pittsburgh Sleep Quality Index	x	x	x	x		
EQ-5D-3L	x	x	x	x		
Service satisfaction	x	x			x	
Perceived usefulness, ease of use, compatibility and usage intention of new AI-based technologies for tailored rehabilitation (TAM)					x*	
ICT skills	x	x	x	x	x	
Health-related out-of-pocket cost issues	x					
Work-related quality of life					x	
Personal data	x	x	x	x	x†	
Diagnostic and healthcare pathways, and the related economic, financial and organisational key performance indicators (KPIs)						x‡

* Healthcare professionals not involved in the study.

† Data on working experience, etc.

‡ Related to the delivery of diagnosis and rehabilitation services for UCP.

EQ-5D-3L, European Quality of Life 5 Dimensions 3 Level Version; ICT, Information & Communication Technologies; TDC, typically developing children; UCP, unilateral cerebral palsy.

### Clinical assessments for typically developing children

TDC will be assessed using the AHA while wearing the two sensors at both wrists that track UpL activity and will be required to keep the sensors on their wrists for the following 14 days, following the same procedure as participants with UCP.

In addition to this, and with the aim of assessing the absence of motor impairments and the hand dominance, their parents will be asked to fill in (i) the Developmental Coordination Disorder Questionnaire (DCDQ),^56^ a brief parent questionnaire designed to screen for coordination disorders in children aged 5–15 years with a 5-point Likert scale. The DCDQ provides a standard method to measure a child’s coordination in everyday functional activities. The internal consistency of the DCDQ is high, and the results from discriminant function analyses were appropriately strong for a screening tool; and (ii) the Edinburgh Handedness Inventory (EHI) questionnaire,^57^ a measurement scale used to assess the dominance of a person’s right or left hand in everyday activities, sometimes referred to as laterality. The inventory can be used by an observer assessing the person or by a person self-reporting hand use. The EHI is a simple quantitative method of assessing handedness, composed of 10 items (eg, writing, drawing, throwing and using scissors).

### Sample size

For this observational study, we plan to collect data from 250 children with UCP. Each clinical site will recruit a certain number of participants: Italy (n=80), Spain (n=70), Belgium (n=50) and Georgia (n=50). In literature, it is recommended for the development of logistic regression models that the smallest predictive outcome group should include at least 100 participants.^58^ Based on a systematic study on sample size prediction for classification, an initially annotated dataset of 100–200 participants is recommended.^59^ Further, in the study by Reid *et al,*^52^ the power analysis revealed that a sample size of 141 is required for a cohort of children with UCP to produce a linear model able to correctly detect a relationship between all scored anatomies (when not discarding any region on the semi-quantitative scale template by Fiori *et al*^50^ and AHA score, corresponding to an overall of 0.15 for the model), with a power of 80% and a statistical significance of α=0.05. As the aim of this study is to build a dDST for providing a valid and reliable functional diagnosis of children with UCP and enable accurate stratification using multiaxial assessments and outcome variables, we aim to include 250 participants with UCP, ensuring robustness of results. In addition, 250 age-matched TDC, with the same distribution of children across sites as with UCP (ie, n=80 for Italy, n=50 for Belgium, n=70 for Spain and n=50 for Georgia) will be recruited to serve as control participants.

### Study outcome

The expected primary outcome of this study is the development of an integrated dDST for UpL functional profiling in children with UCP. The dDST will integrate multidimensional clinical, neuroimaging and digital biomarkers to identify functional profiles and provide information to support personalised rehabilitation decision-making. The clinical validity, generalisability and effectiveness of the resulting recommendations will require prospective validation in subsequent studies.

### Enrollment procedures

Children diagnosed with UCP will be identified by their treating clinicians, researchers or referred to the research team by participating site specialists during their hospitalisation, outpatient follow-up visits or annual developmental re-evaluations. Once identified, study flyers or verbal information about the study will be provided to the family, which will be invited also on exploring the AInCP website (https://www.aincp.eu/). Flyers and information sheets regarding the study will also be circulated within referral centres, clinics, educational facilities, relative associations or foundations. Additionally, information sessions towards parents or associations for children with disabilities will be given to maximise study visibility and convey the study’s impact. Direct contact of the researchers to the families’ will always occur in agreement with the child’s treating clinicians.

For the TDC group, clinical partners will start with a convenient sample of volunteers, while flyers and information sheets regarding the study will also be circulated within mailing lists (ie, schools and leisure or extracurricular activity centres).

Detailed explanation of the study procedures and consent will be given to parents and eligible children by researchers who are not directly involved in the children’s clinical care and follow-up. During this contact, participants will be assured that their participation in the study is voluntary and that their decision about participating in the study will not compromise their child’s current or future care. The importance of the database and the data used for the study will also be explained, for which the request for release for video recording and compilation of the database will also be specified, under an identification code as per the pseudonymisation guidelines of the General Data Protection Regulation (GDPR).

Parents will have enough time to review the information and the opportunity to ask any questions about the study and decide their involvement in the study. If more time is needed, a subsequent conversation will ensure in which the parents will be able to ask clarifying questions and definitively confirm their consent to the study.

Individual written informed consent and informed assent forms will be obtained from parents of all participants and from participants aged >12, respectively. Information regarding the study design, its duration, conduction manner, data collection, data policy and database preparation processes will be explained to the families. Then, consents from parents/legal guardians informed will be collected, ensuring anonymity and the use of the subject-specific code (ID) for the collected data. The nature of the study will be explained also to the children/teenagers using a language suitable to the age range. Interpreters will be sought for parents speaking languages other than the official country language.

The enrolment will commence after informed consent signing; thereafter, families of children with UCP will be contacted to plan a clinical assessment made of two consecutive days, organised in the referral clinical centre (FSM, KU Leuven, UCLM and DTMU). All data acquired will be scored by experts and will be inserted in a REDCap database, specifically designed for the project.

The study has been recruiting participants since February 2024 and is expected to be completed by May 2027.

### Patient and public involvement

The AInCP project adopts a multi-stakeholder approach in which people with living experience are key stakeholders. The involvement of this group is primarily coordinated and supervised by the Fight the Stroke association in Italy. Partner associations, including Hemiweb in Spain and CPinfo in Belgium, are also actively involved, which contributes to dissemination activities and providing structured feedback.

Patient and public involvement has been embedded from the early stages of the AInCP project. Specifically, Fight the Stroke conducted surveys among the target population and provided insights that were instrumental in shaping the project design. Parents, as well as children, share feedback on the study design, relevance and importance of the project goals, burden of study procedures and suitability of materials provided to participants. Furthermore, dissemination activities are officially led by Fight the Stroke, ensuring that all communication materials are tailored to the needs, preferences and expectations of the target population.

### Anticipated challenges

- Selection bias: we will focus on creating the most possible balanced dataset including 250 children with UCP with specific inclusion criteria and stratification (eg, at least 80 children for each MACS level (I–III); at least 80 in each age range, ie, 5–7, 8–12 and 13–15 years) that will be representative of the functional spectrum of the UCP population. In addition, particular attention will be paid to recruiting different (ie, balanced among themselves) patients to provide an inclusive and efficient system.
- Unavailability of brain MRI: since children and/or adolescents will be enrolled in at least the school age, they will more likely have already been tested during their diagnosis with MRI. Differences in image quality due to the discrepancy in scanners across clinical sites will be managed by harmonisation analyses.
- Technical problems regarding the wearable trackers that perform UpL movement analysis during clinical assessment and daily life: participants will be guaranteed timely technical interventions for maintenance and, if necessary, the replacement of trackers.
- Age, sex, gender preferences will account for potential biases and discriminations, using AI algorithms and other statistical methods (eg, models including covariance).
- Cross-sectional assessment at a single study time point: this does not allow assessment of longitudinal change or direct evaluation of the effectiveness of rehabilitation recommendations. Therefore, the dDST developed in this study will require prospective external validation before its clinical recommendations can be considered evidence of diagnosis and treatment effectiveness.

### Data collection

A pseudonymised individual study ID code will be assigned to all participants. At the time of the data analysis, access to the full Patient Identification Record (PIR) will be restricted; in particular, the PIR will be stored separately, with password-protected access for the local principal investigators. All data will be digitalised and stored pseudonymised in the REDCap platform of the Green Data Centre located in UNIPI, which can only be accessed by the involved researchers/clinicians, as agreed in the Joint Controllership Agreement.

Personal, demographic and clinical data deriving from clinical evaluations of each enrolled participant will be collected and entered into REDCap, which will serve as an electronic Case Report Form (eCRF). REDCap will be specifically developed based on the project’s requirements. It is the involved researcher’s responsibility to ensure that all data specified in the AInCP protocol are accurately and completely inserted in REDCap, following instructions provided by the associate platform developers. Paper documentation, if any, will be securely stored at the respective academic institutions, with restricted access only to the local principal investigators and the personnel involved in data acquisition. The REDCap will be developed by the sponsor (UNIPI), and each clinical site will receive a unique password, allowing individual access to their data only.

Based on an average completion time for each assessment, the total protocol administration duration will last approximately 6–8 hours for children with UCP. To avoid participant fatigue, the assessment session will be split in 2/3 days, with assessments in each day lasting around 3–4 hours. A maximal 14-day window will be allowed between the execution of the two assessments, if evaluation during two/three consecutive days is not possible. Since TDC will be assessed with less outcome measures, their total assessment session will last around 45–60 min. Short breaks in-between assessments on the same day will be foreseen. An additional 45–60 min are required for the eHTA survey to be completed, based on the number of questionnaires that each stakeholder must fill in.

Each child will be administered the tests in an identical manner to ensure standardisation. There is the possibility of using lexical terms suitable for the child without changing the nature of the instructions. The child and/or teenager can ask for clarifications at any time. A common assessment manual has been created and shared among the four clinical sites (ie, Italy, Belgium, Spain and Georgia) to ensure smooth research conduct. To corroborate adherence to the protocol, shared material will be established at the clinical centres, mainly composed of an electronic checklist for all data and evaluations to avoid data loss.

### Data analysis plan

As a primary aim, this project envisages the creation of a diagnostic tool that can make diagnosis as personalised as possible. Novel techniques, such as AI and Big Data analytics, could analyse data from large datasets and multiple modalities and will be crucial for the development of the dDST, which will combine the most important factors that delineate UpL function of children with UCP. Since data analysis will involve AI methods, it will be carried out according to AIFA Guidelines (‘Guide to the submission of a request for authorisation of a Clinical Trial involving the use of AI or Machine Learning (ML) systems’, 2021). Data collected at the UNIPI Green Data Centre will be of different nature: from neuroimages’ analysis to clinical assessments to wearable trackers’ data.

UNIPI will process all data using proprietary AI algorithms and Big Data techniques, implemented through tools such as R, Python, PyTorch and Scikit-learn. The project will analyse interdependencies across data domains using clustering, classification and both classical and advanced AI methods, including deep-learning, machine-learning and reinforcement-learning algorithms (eg, support vector machine, k-nearest neighbours, convolutional neural networks, recurrent neural networks and Q-Learning). Big Data analytics will also handle data storage and processing where learning is not required.

All data will be integrated and analysed to generate insights for personalised clinical profiling. Results will be visualised and discussed with clinicians to identify the most meaningful correlations. Statistical analyses will also be conducted across clinical, social, economic and technological data to assess outcomes, costs and quality of life impacts for patients, families, therapists and clinicians. Although no new algorithms will be created, standard ones will be combined to build new and innovative analytical pipelines to support personalised healthcare insights.

For the neuroimaging data, quantitative neuroimaging measures (volumes and cortical shape) will be associated with multiple clinical measures using least absolute shrinkage and selection operator (LASSO regression), which reduces the number of measures to an optimal subset that is relevant for the clinical outcome. LASSO models will be trained on a 75% partition of the data and tested on the remaining 25% test set to validate its predictive performance. Within the training set, cross-validation will be performed to determine the optimal sparsity parameter. Participant age, sex and study site will be included as covariates in all models. One model will be trained for each of the clinical assessments (ie, AHA, WPPSI-IV/WISC-V, NEPSY-II and Beery-VMI).

The overall analysis design for internally evaluating the performance of the dDST will be structured to ensure robustness and minimise bias, given the sample size estimates (~140) and the involvement of multiple sites. To achieve this, a data-splitting strategy will be implemented, in which a proportion of participants will be systematically allocated to the testing subset, ensuring a uniform distribution of data over time and across sites for both training and validation datasets. This systematic split will help mitigate potential biases that could arise from non-random sampling or temporal trends in data collection. Ultimately, the multiple clinical and digital biomarkers (ie, clinical multiaxial assessments, neuroimaging and UpL daily movement analysis) will be combined for the dDST.

### Data ownership

Within the context of an international collaboration (Horizon Europe), data will be owned by the party that generates them. Data that derive from all the clinical assessments, neuroimaging techniques, wearable trackers and eHTA survey and have been acquired by the subsamples of the clinical centres involved shall belong to the respective clinical sites. Overall, five joint data controllers (ie, UNIPI, FSM, UCLM, KU Leuven and DTMU) have been contractually appointed and are bound through a Joint Controllership Agreement. Further, project collaborators have been appointed as data processors, based on their expertise, and are bound through a Data Processing Agreement.

### Audits/inspections

The involved researchers will permit study-related monitoring, audits, institutional review boards/ethical committee reviews and inspections from regulatory authorities, providing direct access to all source data, relevant documentation and study records.

## Ethics and dissemination

The study will be conducted in compliance with the fundamental principles of dignity and human rights and ethical principles, whose sources are among others the Declaration of Helsinki and the Oviedo Convention. The study will be compliant with all applicable international regulations such as the GDPR. Planning and conducting clinical investigations with medical devices will also follow the Code of Good Clinical Practice (UNI EN ISO 14155:2012 STANDARD).

Full ethical approval has been obtained from the Tuscany Paediatric Ethics Committee (number 88/2023), Ethical Committee Research UZ/KU Leuven (S67861) Toledo Hospital Ethics Committee (1068), Ethical Committee of David Tvildiani Medical University (5/23) and CSIRO Health and Medical Human Research Ethics Committee (2022_047_LR). Any deviations from the protocol will be promptly notified to this Ethics Committee and applied only after its approval. The study has been registered at http://www.clinicaltrials.gov (identifier NCT06073522). This study protocol is reported according to the SPIRIT statement^60^ and guidelines.^61^ All procedures will be closely monitored by ethicists (University of Salento, Italy) during AI implementation to ensure patient privacy, prevent bias and avoid unintended harm while developing robust, fair, and trustworthy AI solutions. Signed informed consent will be obtained from caregivers and participants.

Communication material is being developed and will be distributed during structured information sessions and events to the target populations (ie, families with living experience and healthcare providers). Further, dissemination activities involve publications in selected open-access peer-reviewed journals, presentations at scientific conferences, with training and workshops dedicated to rehabilitation specialists. Informative sessions for family associations will be supported and organised in all countries, with the collaboration of the clinical centres. Networking activities will be put in place to involve organisations at national and EU level. Their feedback will be important to contribute to policy shaping and supporting new policy initiatives and decisions.
