# Supplementary material for: Artificial Intelligence for developing a personalised functional profile and supporting upper-limb rehabilitation decision-making in children with unilateral Cerebral Palsy (AInCP): protocol for an observational study
Source: BMJ Open. 2026 Nov 2;16(11):e117833. doi: 10.1136/bmjopen-2026-117833 (PMC13735316; doi:10.1136/bmjopen-2026-117833)
Supplement: Supplementary file 1 [file bmjopen-2026-117833.reviewer_comments.pdf]

PEER REVIEW HISTORY

BMJ Open publishes all reviews undertaken for accepted manuscripts. Reviewers are asked to complete a checklist review form and are provided with free text boxes to elaborate on their assessment. These free text comments are reproduced below.

ARTICLE DETAILS

Title (Provisional)

Artificial Intelligence for developing a personalised functional profile and supporting upper-limb rehabilitation decision-making in children with unilateral Cerebral Palsy (AInCP): protocol for an observational study

Authors

Sgandurra, Giuseppina; Kalkantzi, Alexandra; Beani, Elena; Filogna, Silvia; Cioni, Giovanni; Palomo Carrión, Rocio; Feys, Hilde; Tatishvili, Nana Nino; Cianchetti, Matteo; Battaglia, Fiorella; Turchetti, Giuseppe; Van Dommelen, Wil; Pirini, Marco; Fedeli, Francesca; Bombonato, Clara; Di Lieto, Maria Chiara; Del Lucchese, Benedetta; Barzacchi, Veronica; Menici, Valentina; Cotardo, Annalisa; Rizzo, Marta; Bieber, Eleonora; Gatti, Pietro Ilio; Camuncoli, Federica; Tosetti, Michela; Bosco, Paolo; Biagi, Laura; Peretti, Luca; Cruz-Sanabria, Francy; Lirio-Romero, Cristina; Coello-Villalón, María; López-Muñoz, Purificación; Ángel Basco-López, Julián; Romay Barrero, Helena; Mailleux, Lisa; Tatishvili, Sofia; bedoshvili, anna; Valishvili, Nino; Maselli, Martina; De Luca, Giovanna; Claverini, Corrado; Trieste, Leopoldo; Manfroni, Giorgio; Bult, Harold; Berghold, David; Jakob, Iris; Pagnozzi, Alex Michael; Fripp, Jurgen; Pannek, Kerstin; Ortibus, Els; Scrocco, Antea; D'alessandro, Gemma; Arras, Giovanni; Turchi, Tommaso; Ferragina, Paolo; Boyd, Roslyn N; Prencipe, Giuseppe

---

VERSION 1 - REVIEW

**Reviewer**	**1**
**Name**	**Becher, JG**
**Affiliation**	**VU University Medical Center , Amsterdam, rehabilitation medicine**
**Date**	**06-Aug-2026**
**COI**	**None**

I think that answers on my raised questions will improve the manuscript and useful for the final analysis of the data after finishing the study.

**

This manuscript is the description of a prospective multi-center observational study protocol to obtain a personalized functional diagnosis and tailored treatment strategies with the aid of AI and Big

Data computer analysis for children with unilateral spastic Cerebral Palsy, focussed on the upper extremity.

Four European centers for pediatric rehabilitation will participate (in Belgium, Georgia, Italy, and Spain).

A standard assessment protocol will be performed only at one time point in 250 children with unilateral Cerebral Palsy and 250 typically developing children.

Patients are selected based on at least some motor function of the upper extremity (MACS level III or less) and no severe cognitive or behavioral problems. Also impeding other medical conditions are excluded.

In the protocol, an extensive list of clinical testing is listed, partly based on physical examination of impairments and capacity testing of activities, partly based on questionnaires.

Besides this clinical testing, a MRI-brain scan (T2 technique focussed on white matter brain abnormalities) will be performed.

As an objective measurement, 14 days monitoring with 2 inertial sensors will be performed to measures the use of the affected upper limb in daily life.

To get information from the stakeholders (professionals, parents) a large set of questionnaires will be used about attitude to technique, social circumstances, sleep, quality of life.

Also, in typically developing children the monitoring with 2 upper limb sensors will take place, and some questionnaires about their level of motor performance.

Direct expected outcome of this study is the development and fostering of a newly advanced and integrated decision support tool for the UpL functioning of children with UCP, able to provide a functional diagnosis (dDST) that will allow a better prediction, opening the frontiers of the personalized medicine in child neurology.

GENERAL REMARKS

1. The title of the study suggests that ALL children with unilateral CP are involved. However, the study focusses on mild to moderate involved children. That should be mentioned.

2. Clinical classifications, like MACS, are NOT assessment tools, little sensitive for change: this is a point of interest for the test.

3. Five different countries are participating, most tools are observational tools and questionnaires. Nothing is mentioned about cross-cultural validation and language problems. Are there any assessments done to check the uniformity of the results between countries?

4. There is only one time point for assessments. There are very many variables of one assessment of a child and the environment. I cannot imagine that advises for therapy can be given, as there is no outcome assessment. I presume that based on all data clusters of impairments can be identified. Therapy advise can be given based on these clusters, but there is no evidence for the efficacy and reliability of these advises. How will this research deal with this problem?

VERSION 1 - AUTHOR RESPONSE

Reviewer: 1

This manuscript is the description of a prospective multi-center observational study protocol to obtain a personalized functional diagnosis and tailored treatment strategies with the aid of AI and Big Data computer analysis for children with unilateral spastic Cerebral Palsy, focussed on the upper extremity.

Four European centers for pediatric rehabilitation will participate (in Belgium, Georgia, Italy, and Spain).
A standard assessment protocol will be performed only at one time point in 250 children with unilateral Cerebral Palsy and 250 typically developing children.

Patients are selected based on at least some motor function of the upper extremity (MACS level III or less) and no severe cognitive or behavioral problems. Also impeding other medical conditions are excluded.
In the protocol, an extensive list of clinical testing is listed, partly based on physical examination of impairments and capacity testing of activities, partly based on questionnaires.

Besides this clinical testing, a MRI-brain scan (T2 technique focussed on white matter brain abnormalities) will be performed.

As an objective measurement, 14 days monitoring with 2 inertial sensors will be performed to measures the use of the affected upper limb in daily life.

To get information from the stakeholders (professionals, parents) a large set of questionnaires will be used about attitude to technique, social circumstances, sleep, quality of life.

Also, in typically developing children the monitoring with 2 upper limb sensors will take place, and some questionnaires about their level of motor performance.

Direct expected outcome of this study is the development and fostering of a newly advanced and integrated decision support tool for the UpL functioning of children with UCP, able to provide a functional diagnosis (dDST) that will allow a better prediction, opening the frontiers of the personalized medicine in child neurology.

We sincerely thank the Reviewer for the careful reading of our manuscript and for the constructive and insightful comments. We agree that addressing these points will improve the clarity of the study scope, the interpretation of the outcome measures, and the description of the multicentre methodology. We have therefore revised the manuscript accordingly.

GENERAL REMARKS

1. The title of the study suggests that ALL children with unilateral CP are involved. However, the study focusses on mild to moderate involved children. That should be mentioned.

We thank the reviewer for this insightful comment. We appreciate the opportunity to clarify our rationale regarding the inclusion criteria and the study title. In our study, we included children with unilateral cerebral palsy (uCP) classified within MACS levels I to III. As documented in the literature, children with uCP almost exclusively fall within MACS levels I–III because the presence of a fully functional, non-involved hand prevents the need for the continuous, extensive assistance characteristic of levels IV and V (Eliasson et al., 2006; Lidman et al., 2023). This is also supported by the fact that standard upper-limb assessments for children with uCP such as the Assisting Hand Assessment (AHA) are validated specifically for children functioning at MACS levels I–III. Since MACS levels I to III encompass virtually the entire spectrum of manual ability observed in the unilateral CP population, we believe that the title is representative of this cohort. However, to ensure maximum clarity for the readers, we have mention this distribution in the MS.

References:

- *Eliasson AC, et al. (2006). The Manual Ability Classification System (MACS) for children with cerebral palsy: scale development and evidence of validity and reliability. BMC Musculoskelet Disord, 7:50. (DOI: 10.1186/1471-2474-7-50)*
- *Lidman G, et al. (2023). Developmental trajectories of hand function in children with unilateral cerebral palsy. Dev Med Child Neurol. (DOI: 10.1111/dmcn.15370)*

2. Clinical classifications, like MACS, are NOT assessment tools, little sensitive for change: this is a point of interest for the test.

Thanks. We agree with this observation and apologize for any lack of clarity. In our study, the MACS will be used exclusively to characterize and stratify the study population, rather than as a quantitative outcome measure or an indicator of intervention-induced change. This aspect has now been clarified in the revised MS.

3. Five different countries are participating, most tools are observational tools and questionnaires. Nothing is mentioned about cross-cultural validation and language problems. Are there any assessments done to check the uniformity of the results between countries?

We thank the Reviewer for raising this important methodological issue. We would first like to clarify that the study involves four clinical centres in four countries: Italy, Belgium, Spain and Georgia. Australia is involved in the analysis of part of the neuroimaging data but is not a clinical recruitment centre.
To ensure consistency across the four clinical centres, a common assessment manual has been developed and shared among all sites. The protocol specifies that assessments will be administered in an identical manner, with standardized instructions and shared materials, and that an electronic checklist will be used to monitor adherence to the assessment protocol and completeness of data collection.

We are also aware of the potential impact of linguistic and cross-cultural differences on the assessment of clinical and behavioural outcomes. Whenever available, we used standardized instruments with validated versions in the respective languages of the participating countries. For instruments for which a validated version was not available, the translated versions were prepared using a back-translation procedure to ensure semantic and conceptual equivalence with the original version, and this has mainly been done for tests in Georgian.

Furthermore, for outcome measures for which cross-country differences may potentially affect the results, the statistical analyses will specifically take country/site into account, and appropriate sensitivity or subgroup analyses will be performed where warranted. In practice, these considerations are expected to be particularly relevant to the Georgian site, where some translated versions of the instruments are currently being further evaluated. Stating the effort of providing translated versions, we are also

considering a dedicated validation study of these translated versions, which would provide additional evidence regarding their linguistic and psychometric equivalence to the original instruments. Together, these procedures were implemented to maximize the comparability and consistency of assessments across participating countries while acknowledging and appropriately addressing potential cross-cultural and linguistic differences.

4. There is only one time point for assessments. There are very many variables of one assessment of a child and the environment. I cannot imagine that advises for therapy can be given, as there is no outcome assessment. I

presume that based on all data clusters of impairments can be identified. Therapy advise can be given based on these clusters, but there is no evidence for the efficacy and reliability of these advises. How will this research deal with this problem?

We agree that the single time-point observational design does not allow us to evaluate the efficacy of rehabilitation recommendations. The purpose of the current study is instead to identify multidimensional functional profiles and develop the dDST.

The primary purpose of the AInCP observational study is to develop and internally validate a multidimensional functional profiling and diagnostic Decision Support Tool (dDST) by integrating clinical assessments, neuroimaging and upper-limb daily-life monitoring. The intention is to identify relationships among these domains and to characterize clinically meaningful functional profiles that may inform individualized rehabilitation planning. We acknowledge that the original wording of the manuscript could be interpreted as implying that the present study will directly establish the efficacy of AI-derived treatment recommendations. This was not our intention. We have therefore revised the manuscript to distinguish clearly between: i) identification of multidimensional functional profiles and rehabilitation needs, which is an objective of the present observational study; and ii) validation of treatment recommendations and their efficacy, which will require subsequent prospective longitudinal and/or interventional studies. Accordingly, the dDST generated from the present study should be considered a clinical decision-support and hypothesis-generating tool, rather than a validated treatment recommendation system. The present dataset will provide the basis for identifying candidate biomarkers and functional clusters and for generating personalized hypotheses regarding rehabilitation needs. These hypotheses will need to be prospectively tested in future studies, including longitudinal assessment of treatment response. In particular, we now emphasize that the AInCP study aims to provide a personalized functional diagnosis and to inform or support future individualized treatment planning, while acknowledging that the clinical effectiveness and reliability of such recommendations require subsequent validation.

*** ***

COI statement(s):
Reviewer: 1

If you have selected ‘Yes’ above, please provide details of any competing interests.: Not applicable.
